# Supplementary material for: Perpetration and Victimization in Offline and Cyber Contexts: A Variable- and Person-Oriented Examination of Associations and Differences Regarding Domain-Specific Self-Esteem and School Adjustment
Source: Int J Environ Res Public Health. 2021 Oct 3;18(19):10429. doi: 10.3390/ijerph181910429 (PMC8508291; doi:10.3390/ijerph181910429)
Supplement: Supplementary file 1 [file ijerph-18-10429-s001.zip › ijerph-1381639-supplementary.pdf]

Supplemental Material

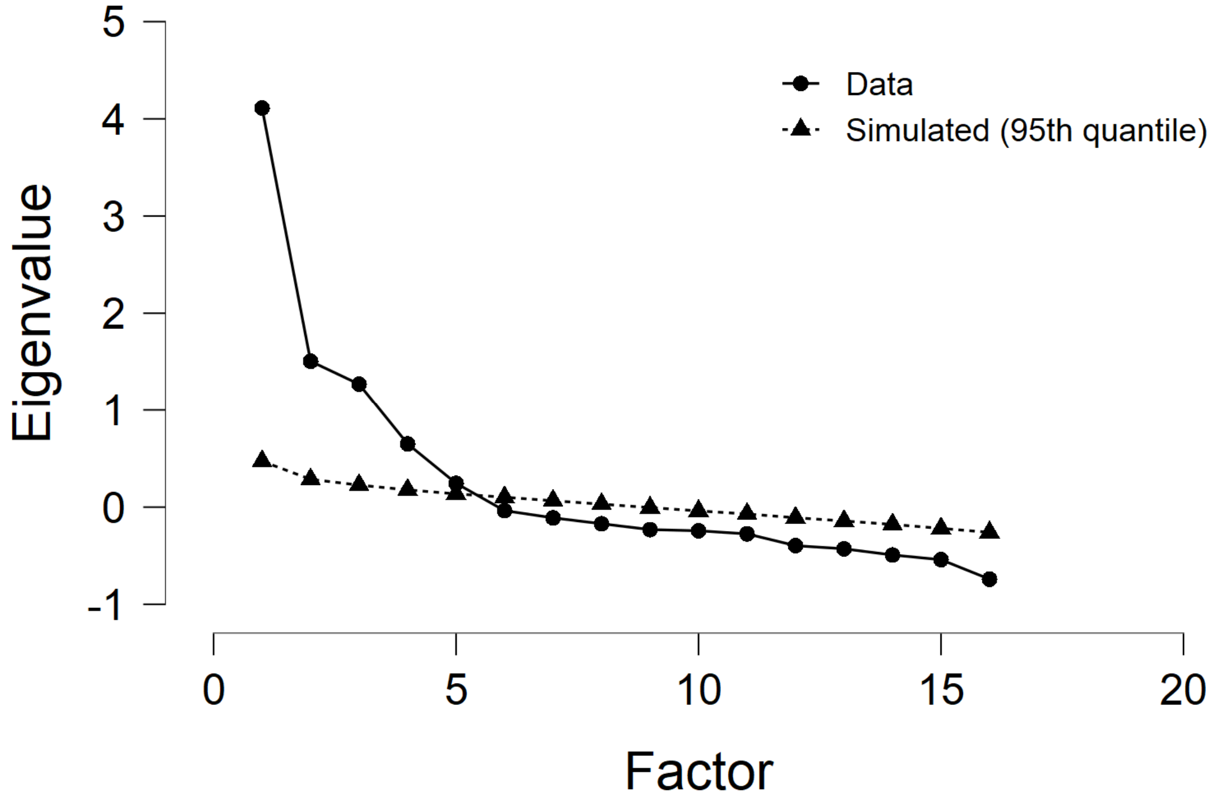

Figure S1. Scree Plot of Empirical Eigenvalues Against the 95th Quantile of Simulated Eigenvalues.

Supplementary Table S1. Unstandardized Parameters of the Mediation Model.

						95% CI	
**Predictors**	**Outcome**	**Estimate**	***SE***	***z***	***p***	**Lower**	**Upper**
school adjustment	offline victimization	**−0.465****	0.149	−3.129	0.002	−0.757	−0.174
school adjustment	offline perpetration	0.195	0.211	0.925	0.355	−0.218	0.608
school adjustment	cyber victimization	0.144	0.225	0.640	0.522	−0.297	0.585
school adjustment	cyber perpetration	−0.670	0.474	−1.412	0.158	−1.600	0.260
school adjustment	school performance SE	**0.247*****	0.074	3.342	< 0.001	0.102	0.392
school adjustment	body related SE	0.065	0.058	1.128	0.259	−0.048	0.178
school adjustment	social SE	**0.573*****	0.085	6.710	< 0.001	0.406	0.741
school adjustment	creative−artistic SE	−0.069	0.055	−1.262	0.207	−0.177	0.038
school adjustment	emotional SE	**0.579*****	0.087	6.676	< 0.001	0.409	0.749
offline victimization	school performance SE	**0.175*****	0.040	4.353	< 0.001	0.096	0.254
offline victimization	body related SE	−0.070	0.040	−1.729	0.084	−0.148	0.009
offline victimization	social SE	**−0.328*****	0.048	−6.810	< 0.001	−0.423	−0.234
offline victimization	creative−artistic SE	0.025	0.036	0.683	0.494	−0.046	0.095
offline victimization	emotional SE	−0.090	0.049	−1.828	0.067	−0.187	0.006
offline perpetration	school performance SE	−0.079	0.046	−1.724	0.085	−0.168	0.011
offline perpetration	body related SE	0.006	0.037	0.157	0.876	−0.067	0.079
offline perpetration	social SE	−0.008	0.033	−0.253	0.800	−0.073	0.056
offline perpetration	creative−artistic SE	0.030	0.024	1.244	0.214	−0.018	0.078
offline perpetration	emotional SE	−0.029	0.035	−0.811	0.418	−0.098	0.041
cyber victimization	school performance SE	**0.054***	0.025	2.187	0.029	0.006	0.102
cyber victimization	body related SE	0.011	0.027	0.407	0.684	−0.041	0.063
cyber victimization	social SE	**−0.155*****	0.029	−5.280	< 0.001	−0.213	−0.097
cyber victimization	creative−artistic SE	−0.0001524	0.022	−0.007	0.994	−0.043	0.043
cyber victimization	emotional SE	−0.043	0.029	−1.453	0.146	−0.100	0.015

cyber perpetration	school performance SE	−0.065	0.038	−1.703	0.088	−0.139	0.010
cyber perpetration	body related SE	0.036	0.031	1.159	0.246	−0.025	0.096
cyber perpetration	social SE	−0.004	0.019	−0.207	0.836	−0.042	0.034
cyber perpetration	creative−artistic SE	−0.011	0.018	−0.594	0.553	−0.047	0.025
cyber perpetration	emotional SE	−0.029	0.023	−1.274	0.203	−0.073	0.016
school performance SE	gender	−0.231	0.179	−1.286	0.199	−0.582	0.121
school performance SE	age	−0.031	0.033	−0.946	0.344	−0.095	0.033
body related SE	gender	−0.070	0.189	−0.373	0.709	−0.440	0.299
body related SE	age	0.038	0.037	1.023	0.306	−0.035	0.111
social SE	gender	0.112	0.167	0.669	0.503	−0.216	0.440
social SE	age	0.017	0.034	0.510	0.610	−0.050	0.085
creative−artistic SE	gender	−0.295	0.205	−1.434	0.152	−0.697	0.108
creative−artistic SE	age	0.010	0.036	0.289	0.773	−0.060	0.080
emotional SE	gender	**0.390***	0.169	2.309	0.021	0.059	0.720
emotional SE	age	0.025	0.035	0.702	0.482	−0.044	0.093
offline victimization	gender	0.126	0.088	1.436	0.151	−0.046	0.297
offline victimization	age	0.017	0.017	1.038	0.299	−0.015	0.050
offline perpetration	gender	**0.220****	0.072	3.042	0.002	0.078	0.361
offline perpetration	age	0.002	0.010	0.191	0.849	−0.018	0.022
cyber victimization	gender	0.036	0.054	0.658	0.510	−0.070	0.142
cyber victimization	age	−0.0002317	0.010	−0.023	0.982	−0.020	0.019
cyber perpetration	gender	0.060	0.035	1.712	0.087	−0.009	0.128
cyber perpetration	age	0.004	0.004	1.003	0.316	−0.004	0.012
school adjustment	gender	0.231	0.133	1.739	0.082	−0.029	0.491
school adjustment	age	−0.045	0.025	−1.762	0.078	−0.094	0.005

Note. SE = "self-esteem". Robust standard errors, robust confidence intervals, full information maximum likelihood estimator. Significant values are displayed in bold. * $p \leq 0.05$, ** $p \leq 0.01$, *** $p \leq 0.001$.

Supplementary Table S2. Unstandardized Total, Direct, Total Indirect and Indirect Effects of the Mediation Model.

							95% CI	
**Predictors**	**Mediator**	**Outcome**	**Estimate**	***SE***	***z***	***p***	**Lower**	**Upper**
**Total effects**								
school performance SE	—	school adjustment	**0.201****	0.073	2.753	0.006	0.058	0.345
body related SE	—	school adjustment	0.076	0.063	1.210	0.226	−0.047	0.200
social SE	—	school adjustment	**0.705*****	0.077	9.184	≤ 0.001	0.554	0.855
creative−artistic SE	—	school adjustment	−0.068	0.057	−1.196	0.232	−0.179	0.043
emotional SE	—	school adjustment	**0.629*****	0.091	6.879	≤ 0.001	0.449	0.808
**Direct effects**								
school performance SE	—	school adjustment	**0.247*****	0.074	3.342	≤ 0.001	0.102	0.392
body related SE	—	school adjustment	0.065	0.058	1.128	0.259	−0.048	0.178
social SE	—	school adjustment	**0.573*****	0.085	6.710	≤ 0.001	0.406	0.741
creative−artistic SE	—	school adjustment	−0.069	0.055	−1.262	0.207	−0.177	0.038
emotional SE	—	school adjustment	**0.579*****	0.087	6.676	≤ 0.001	0.409	0.749
**Total indirect effects**								
school performance SE	—	school adjustment	−0.046	0.047	−0.978	0.328	−0.137	0.046
body related SE	—	school adjustment	0.011	0.035	0.319	0.750	−0.057	0.080
social SE	—	school adjustment	**0.131*****	0.040	3.254	0.001	0.052	0.210
creative−artistic SE	—	school adjustment	0.002	0.021	0.081	0.935	−0.040	0.043
emotional SE	—	school adjustment	**0.050***	0.025	1.962	0.050	0.00006	0.099
**Indirect effects**								
school performance SE	offline victimization	school adjustment	**−0.082***	0.032	−2.557	0.011	−0.144	−0.019
school performance SE	offline perpetration	school adjustment	−0.015	0.018	−0.862	0.389	−0.050	0.020
school performance SE	cyber victimization	school adjustment	0.008	0.013	0.616	0.538	−0.017	0.032
school performance SE	cyber perpetration	school adjustment	0.043	0.047	0.920	0.358	−0.049	0.136
body related SE	offline victimization	school adjustment	0.032	0.023	1.432	0.152	−0.012	0.077
body related SE	offline perpetration	school adjustment	0.001	0.007	0.153	0.878	−0.013	0.016

body related SE	cyber victimization	school adjustment	0.002	0.004	0.379	0.705	−0.006	0.010
body related SE	cyber perpetration	school adjustment	−0.024	0.033	−0.735	0.462	−0.088	0.040
social SE	offline victimization	school adjustment	**0.153****	0.055	2.787	0.005	0.045	0.260
social SE	offline perpetration	school adjustment	−0.002	0.007	−0.250	0.802	−0.014	0.011
social SE	cyber victimization	school adjustment	−0.022	0.035	−0.642	0.521	−0.091	0.046
social SE	cyber perpetration	school adjustment	0.003	0.013	0.205	0.837	−0.023	0.028
creative−artistic SE	offline victimization	school adjustment	−0.011	0.017	−0.662	0.508	−0.045	0.023
creative−artistic SE	offline perpetration	school adjustment	0.006	0.008	0.788	0.430	−0.009	0.021
creative−artistic SE	cyber victimization	school adjustment	−0.00002	0.003	−0.007	0.994	−0.006	0.006
creative−artistic SE	cyber perpetration	school adjustment	0.007	0.015	0.486	0.627	−0.022	0.037
emotional SE	offline victimization	school adjustment	0.042	0.025	1.670	0.095	−0.007	0.091
emotional SE	offline perpetration	school adjustment	−0.006	0.009	−0.606	0.545	−0.024	0.012
emotional SE	cyber victimization	school adjustment	−0.006	0.011	−0.580	0.562	−0.027	0.015
emotional SE	cyber perpetration	school adjustment	0.019	0.019	1.012	0.311	−0.018	0.057

Note. SE = "self-esteem". Robust standard errors, robust confidence intervals, full information maximum likelihood estimator. Significant vales are displayed in bold. * $p \leq 0.05$, ** $p \leq 0.01$, *** $p \leq 0.001$.

Supplementary Table S3. Standardized Total, Direct, Total Indirect and Indirect Effects of the Mediation Model.

							95% CI	
**Predictors**	**Mediator**	**Outcome**	**Estimate**	***SE***	***z***	***p***	**Lower**	**Upper**
**Total effects**								
school performance SE	—	school adjustment	**0.125****	0.045	2.753	0.006	0.036	0.214
body related SE	—	school adjustment	0.053	0.044	1.210	0.226	−0.033	0.139
social SE	—	school adjustment	**0.469*****	0.051	9.184	≤ 0.001	0.369	0.570
creative-artistic SE	—	school adjustment	−0.051	0.043	−1.196	0.232	−0.135	0.033
emotional SE	—	school adjustment	**0.418*****	0.061	6.879	≤ 0.001	0.299	0.537
**Direct effects**								
school performance SE	—	school adjustment	**0.153*****	0.046	3.342	≤ 0.001	0.063	0.243
body related SE	—	school adjustment	0.045	0.040	1.128	0.259	−0.034	0.124
social SE	—	school adjustment	**0.382*****	0.057	6.710	≤ 0.001	0.270	0.493
creative-artistic SE	—	school adjustment	−0.052	0.041	−1.262	0.207	−0.134	0.029
emotional SE	—	school adjustment	**0.385*****	0.058	6.676	≤ 0.001	0.272	0.498
**Total indirect effects**								
school performance SE	—	school adjustment	−0.028	0.029	−0.978	0.328	−0.085	0.028
body related SE	—	school adjustment	0.008	0.024	0.319	0.750	−0.040	0.056
social SE	—	school adjustment	**0.088****	0.027	3.254	0.001	0.035	0.140
creative-artistic SE	—	school adjustment	0.001	0.016	0.081	0.935	−0.030	0.033
emotional SE	—	school adjustment	**0.033***	0.017	1.962	0.050	0.00004	0.066
**Indirect effects**								
school performance SE	offline victimization	school adjustment	**−0.051***	0.020	−2.557	0.011	−0.089	−0.012
school performance SE	offline perpetration	school adjustment	−0.009	0.011	−0.862	0.389	−0.031	0.012
school performance SE	cyber victimization	school adjustment	0.005	0.008	0.616	0.538	−0.010	0.020
school performance SE	cyber perpetration	school adjustment	0.027	0.029	0.920	0.358	−0.030	0.084
body related SE	offline victimization	school adjustment	0.023	0.016	1.432	0.152	−0.008	0.054
body related SE	offline perpetration	school adjustment	0.0008	0.005	0.153	0.878	−0.009	0.011
body related SE	cyber victimization	school adjustment	0.001	0.003	0.379	0.705	−0.005	0.007
body related SE	cyber perpetration	school adjustment	−0.017	0.023	−0.735	0.462	−0.061	0.028
social SE	offline victimization	school adjustment	**0.102****	0.036	2.787	0.005	0.030	0.173
social SE	offline perpetration	school adjustment	−0.001	0.004	−0.250	0.802	−0.010	0.007
social SE	cyber victimization	school adjustment	−0.015	0.023	−0.642	0.521	−0.060	0.031
social SE	cyber perpetration	school adjustment	0.002	0.009	0.205	0.837	−0.015	0.019
creative-artistic SE	offline victimization	school adjustment	−0.009	0.013	−0.662	0.508	−0.034	0.017
creative-artistic SE	offline perpetration	school adjustment	0.004	0.006	0.788	0.430	−0.007	0.016
creative-artistic SE	cyber victimization	school adjustment	−0.00002	0.002	−0.007	0.994	−0.005	0.005
creative-artistic SE	cyber perpetration	school adjustment	0.005	0.011	0.486	0.627	−0.017	0.028
emotional SE	offline victimization	school adjustment	0.028	0.017	1.670	0.095	−0.005	0.061

emotional SE	offline perpetration	school adjustment	−0.004	0.006	−0.606	0.545	−0.016	0.008
emotional SE	cyber victimization	school adjustment	−0.004	0.007	−0.580	0.562	−0.018	0.010
emotional SE	cyber perpetration	school adjustment	0.013	0.013	1.012	0.311	−0.012	0.038

Note. SE = "self-esteem". Robust standard errors, robust confidence intervals, full information maximum likelihood estimator. Significant vales are displayed in bold. * $p \leq 0.05$, ** $p \leq 0.01$, *** $p \leq 0.001$.
